# Effects of Pristine and Aged LDPE and PP Microplastic Leachates on Behavioural Responses of the Soil Arthropods *Folsomia candida* and *Porcellionides pruinosus*

**DOI:** 10.3390/toxics14060502

**Published:** 2026-06-09

**Authors:** Andrea Masseroni, Lorenzo Federico, Alessandro Becchi, Maurizio Quinto, Francesco Saliu, Sara Villa

**Affiliations:** 1 DISAT—Department of Earth and Environmental Sciences, University of Milano-Bicocca, Piazza della Scienza 1, 20126 Milan, Italy; andrea.masseroni@unimib.it (A.M.); lorenzo.federico@unimib.it (L.F.); a.becchi1@campus.unimib.it (A.B.); francesco.saliu@unimib.it (F.S.); 2 DAFNE—Department of Agriculture, Food, Natural Resources and Engineering, University of Foggia, Via Napoli 25, 71122 Foggia, Italy; maurizio.quinto@unifg.it

**Keywords:** soil ecosystem, population behaviour, ecotoxicology, plastic leachates, weathering

## Abstract

This study investigated the behavioural responses of the arthropods *Folsomia candida* (springtails) and *Porcellionides pruinosus* (woodlice) to leachates released from additive-free plastic polymers. Avoidance behaviour was evaluated to assess potential reductions in soil habitat function, while aggregation status was investigated to highlight possible functional impairments in the woodlice population. Leachates from pristine and artificially aged low-density polyethylene (LDPE) and polypropylene (PP) microplastics were tested at three different concentrations, ranging from environmentally relevant levels to a worst-case scenario of soil contamination. The distinct physicochemical structures of LDPE and PP led to different release compounds. The results revealed no statistically significant avoidance responses in arthropods for either treatment. Unlike PP, LDPE induced a statistically significant impairment of gregarious behaviour at the highest tested concentration (150 mg/kg d.w.). Furthermore, pristine LDPE induced more pronounced disaggregation than the aged one, suggesting that weathering may modulate behavioural responses depending on polymer type and endpoint. Therefore, it is recommended that high levels of plastic leachates can have an adverse effect on soil arthropods and that the aggregation behaviour of woodlice may be a more informative and sensitive biological endpoint than avoidance alone.

## 1. Introduction

Microplastics (MP, diameter < 1 mm, according to [1]) are recognised as emerging contaminants that pose a threat to soil health and global food security [2]. The annual amount of MPs entering terrestrial ecosystems has been estimated to be up to 20 times higher than that entering the oceans [3,4]. Among the different ways of entry, plastic equipment of intensive agriculture has been identified as a primary contributing factor [5]. Only in Europe, conventional plastics account for over 98% of agricultural plastics placed on the market, with plastic films representing 76% of total use [6]. Global plastic film demand is expected to rise by 9.5 million tonnes in 2030 [2].

The application of plastic mulch films enhances food security and sustainability since it significantly improves water and nutrient efficiency, thermal insulation, and reduces soil erosion, thus allowing for more efficient pesticide usage [7]. However, the lifespan of on-field mulches is shorter than one year due to the physical and chemical degradation that leads to their fragmentation into MPs and/or nanoplastics [8,9], leading to concentrations ranging from 50 to 260 kg ha^−1^ in long-term cultivated areas [6].

In this context, the application of mulches has been questioned by the scientific community, as it has the potential to exert a significant adverse effect on agroecosystem biodiversity and services [10]. Low-density polyethylene (LDPE) and polypropylene (PP) constitute the primary and most prevalent constituents of fossil mulching films [11]. Recent studies have documented the adverse effects of the MPs on soil organisms, including the induction of oxidative stress, physical damage to the digestive systems, and interference with nutrient assimilation [12,13]. The evaluation of effects induced by small plastic items on organisms is an inherently complex process, as it is influenced by numerous plastic characteristics that must be taken into consideration, including polymer type, shape, size, concentration, and exposure duration [14].

Furthermore, when analysing environmental MP contamination, it is relevant to consider that the vast majority of MPs in agricultural soils are deemed to be aged. In terrestrial systems, degradation pathways are strongly affected by burial conditions, oxygen availability, surface exposure, and previous photo-oxidative history. In particular, agricultural polyethylene films exposed at the soil surface generally exhibit stronger oxidation, increased O/C ratios, chain scission, embrittlement, and progressive loss of mechanical properties compared to buried materials, highlighting the dominant role of photo-oxidation under field conditions [15]. Previous work also demonstrated that preliminary UV ageing promotes the formation of oxygenated functional groups, increases hydrophilicity, and enhances surface erosion, thereby facilitating subsequent degradation processes during soil burial [16]. At the same time, naturally weathered plastics recovered from soils have been shown to develop oxidised surface layers and ageing signatures that may differ from those generated under UV-only laboratory treatments, suggesting that soil ageing involves complex coupled abiotic and biotic mechanisms [17]. Overall, current evidence indicates that plastic degradation in soils is a multifactorial process controlled by the interaction between photo-oxidation, burial scenario, oxygen diffusion, and environmental exposure history, ultimately influencing fragmentation behaviour and the formation of oxygenated degradation products. Weathering causes fragmented shapes, surface roughness, the occurrence of fractures, and alterations in the chemical properties of plastic items [18,19]. In an environmental risk assessment perspective, these features have the potential to considerably influence the toxicity of the polymers. Notwithstanding this, the majority of studies still focus on the impact of pristine MPs, which are readily standardised and comparable but may not be ecologically representative, thereby leading to knowledge gaps on the contribution of weathering in MP toxicity [20].

Another crucial consideration is the evaluation of the role of additives in the onset of MP hazard. Additives (with over 16,000 chemicals, such as plasticisers, flame retardants, stabilisers, and antioxidants) are those potentially toxic compounds typically present during the plastic manufacturing process to shape the material and provide specific functions [21,22]. Once in the environment, plastic additives tend to leach out and migrate into various matrices, possibly leading to further harmful consequences [23]. In the last few years, plastic leachate has started to be studied from an environmental risk assessment perspective. However, the major outcomes suggested that the ecotoxicological implications of plastic leachate are not yet fully understood due to the inherently complex nature of plastic pollution, which widely depends on the plastic type and the preparation methods used for leachates [24]. Moreover, it is challenging to discern the main factors driving the hazard, particularly the contribution of additives compared to the effects induced by polymer residues or the degradation products resulting from exposure to atmospheric agents [25,26].

**Figure 1:**
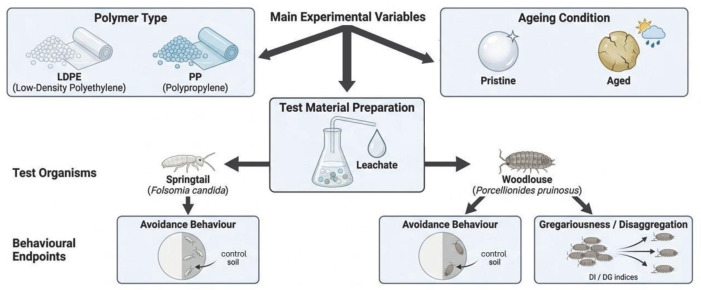
This graphical element highlights the study design. The image shows the different leachates tested in the present study. These were derived from two different polymer types (LDPE and PP) in two different states of ageing (pristine and aged). The effects of the leachates were evaluated by analysing alterations to behavioural endpoints in two terrestrial arthropods (*Folsomia candida* and *Porcellionides pruinosus*). The figure was created by FigureLab and subsequently tailored by the authors (https://www.figurelabs.ai, accessed on 12 September 2025).

To the best of our knowledge, only a few toxicological tests have explored the effects of plastic granule leachate on soil organisms. In this context, the present study aims to offer new insights into the toxicity caused by raw LDPE and PP micronized granules (comparable to additive-free pre-production products) leachates on two terrestrial models, *Folsomia candida* (springtail) and *Porcellionides pruinosus* (woodlice), comparing weathered versus pristine MP leachates (Figure 1). These two model organisms were selected, as they are sensitive to soil contamination [27,28,29,30].

To evaluate the impact of MP leachates, behavioural tests were performed since they are sublethal ecotoxicological bioassays sensitive to soil stressors [31,32], assuming that weathering conditions and polymer type can modulate the responses of arthropods.

According to literature findings, LDPE and PP are in fact expected to follow partially different photo-oxidative degradation pathways due to intrinsic differences in their polymeric structures. In particular, PP contains tertiary carbon atoms that lower the C–H bond dissociation energy compared to polyethylene, thus facilitating radical initiation under UV irradiation and accelerating oxidative degradation processes [33]. Previous experimental studies demonstrated that PP exhibits significantly faster oxidation kinetics than PE during UV exposure [34]. Early stages of PP photo-oxidation are characterised by rapid hydroperoxide accumulation, followed by decomposition into carbonyl compounds and volatile peroxide species, ultimately promoting extensive oxidative chain scission and gaseous product formation [35,36]. In contrast, LDPE degradation is generally described as a more progressive radical auto-oxidation process involving gradual formation of oxygenated functionalities over time [37,38]. Overall, these differences support the hypothesis that these polymers may generate different degradation products and potentially induce distinct environmental effects. These findings may serve as a proxy to explore differences in harmful effects induced by additive-free leachates of polymers as a function of weathering.

## 2. Materials and Methods

### 2.1. Soil Organisms Population Maintenance

Individuals of *Folsomia candida* (Willem, 1902) were cultured in glass Petri dishes (⌀ = 8.5 cm) on a moist substrate of plaster of Paris and activated charcoal (8:1 *w*/*w*), and maintained at a temperature of 20 ± 2 °C, a photoperiod regime of 16:8 h (light:dark), and fed weekly with dried baker’s yeast (*Saccharomyces cerevisiae*). Collembola eggs were periodically separated from the culture to obtain synchronised individuals for tests. Only adults 20 days after hatching were selected.

Individuals of *Porcellionides pruinosus* (Brandt, 1833) culture were maintained in the laboratory at controlled conditions (20 ± 2 °C temperature and photoperiod of 16:8 h of light and dark) and were fed ad libitum with fresh yeast (*Saccharomyces cerevisiae*). The cultures were sprayed with ultra-pure water twice a week, and food was provided. Only adults with a wet weight of 14–30 mg were used during the tests. No sex distinction was performed, as current literature on terrestrial isopod aggregation behaviour identifies environmental conditions and social cues, rather than sex composition, as the primary drivers of aggregation responses [39,40]. Moulting animals, individuals with malformations, and pregnant females were excluded.

### 2.2. Standard Soil

For the toxicity bioassays, the natural LUFA 2.2 sandy loam soil (LUFA Speyer, Speyer, Germany) was used as a standard soil. The properties of this soil include a pH = 5.6 ± 0.3 (0.01 M CaCl_2_), Water Holding Capacity (WHC) = 48.9 ± 5.6 (g/100 g), C = 1.82 ± 0.5 (%), N = 0.19 ± 0.05, Cation Exchange Capacity = 9.54 ± 1.36 meq/100 g.

### 2.3. Preparation and Characterisation of Leachates

Low-density polyethylene (LDPE) and polypropylene (PP) MPs were supplied by Arcoplex (Via Francesca, 1/A-24,040 Pontirolo Nuovo (BG)-Italy) in the form of plastic granulates (150 ± 70 µm in size). The detailed MP characterisation and the description of the artificial weathering procedure are reported in [19]. Briefly, the reference plastic materials were artificially photo-aged by using a QUV accelerated weathering tester (Q-LAB, Saarbrucken, Germany) with UV lamps at 340 nm, with 0.76 W m^−2^ nm^−1^. The ageing test was conducted at 65 °C for a total time of 460 h, with a total UV dose of 170 MJ/m^2^ measured by a bolometer. This dose is equivalent to approximately 10 months of outdoor exposure at 31° N latitude [19]. To prepare the stock leachate solutions, for each treatment, 250 mg of each MP type was weighed, dispersed in 250 mL of deionised water, and placed in a single VELP C4F leaching test batch. The batch was stirred in the dark at 24 ± 1 °C and 90 rpm for 168 h. Each solution was then vacuum filtered through 1.2 µm glass fibre filters to obtain the mother solutions (1 g/L). The final tested concentrations of pristine LDPE (LDPE-P) and PP (PP-P) and of aged LDPE (LDPE-A) and PP (PP-A) were then obtained through serial dilution in deionised water. For each treatment, the tested concentrations were obtained from a single mother solution. Consequently, variation associated with differences between batches could not be assessed. Three nominal concentrations for each MP leachate were chosen (*F. candida* test: 50, 100, and 200 mg/kg d.w.; *P. pruinosus* test: 38, 76, and 152 mg/kg d.w.). These concentrations fall within the estimated environmental concentrations [41,42,43], with high levels ranging from 100 mg/kg d.w. [44] to 500 mg/kg d.w. in the worst-case soil scenario [45]. Negative control samples (blanks) were prepared from 250 mL of deionised water without any plastic added and submitted to the same procedure used for the reference granules and MPs.

Chemical analyses were performed for all batches (LDPE-A, LDPE-P, PP-A, PP-P). The detailed description of leachate characterisation through dissolved organic carbon (DOC), gas chromatography–mass spectrometry (GC–MS) analyses of leachate extracts in two different organic solvents, and the qualitative profiles of the volatile fraction obtained by solid-phase microextraction (HS-SPME) are fully reported in Supplementary Materials (Section S1).

### 2.4. Behavioural Test

Avoidance responses of soil arthropods were performed following the ISO guidelines [46], while the combined avoidance and disaggregation effect was assessed only in woodlice [47]. Glass arenas (9.5 × 7.3 × 5 cm) and glass Petri dishes (⌀ = 100 mm) were used as container tests for *P. pruinosus* and *F. candida*, respectively. Containers were divided into two sides using a removable plastic split and filled with 50 g d.w. of LUFA 2.2 soil on each side. One side was filled with soil contaminated by plastic-leachate solutions, while the other part was filled with soil moistened only with distilled water. The position of the contaminated soil was randomised between the left and right sides. Dual controls were set up using only uncontaminated LUFA 2.2 soil on both sides of the containers. Both dual controls and treatments were moisturised to achieve 40% and 50% of WHC in woodlice and springtails, respectively. All controls and treated soils were gently flattened to obtain a soil surface as homogeneous and lump-free as possible, thereby minimizing thigmotactic effects from the woodlice. For each treatment, ten individuals of *F. candida* and *P. pruinosus* were separately introduced into each arena, with five independent replicates per treatment, and no acclimatation period was applied prior to the start of the bioassay. The organisms were randomly assigned across treatments to avoid any bias. The tests were carried out in thermostatic chambers (WRS UR 700C HPL, Serie WRS UR-KW, KW Apparecchi Scientifici, Monteriggioni, Italy) at 20 ± 1 °C, with a 16:8 h (light:dark) photoperiod, for 48 h. To ensure uniform environmental and lighting conditions across all experimental units, the arenas were placed on the same shelf within the climate-controlled chamber, exposed to the same light intensity, and maintained at the same distance from the light source. During the experiments, the individuals were never fed to reduce aggregation or attraction behaviour induced by food. At the end of the terrestrial isopod test, each arena was gently positioned beneath a camera (Trust Teza 4K Ultra HD webcam, 3840 × 2160 pixels, Trust International BV, Dordrecht, The Netherlands) inside the thermostatic chamber, left undisturbed for 5 min to mitigate any effects that may be induced by vibration resulting from the movement of the arena, and high-resolution colour images were manually recorded for subsequent analysis by ImageJ software (v1.54i). At the end of the springtail test, a plastic divider was placed in the centre of the Petri dish; each soil section was then flooded with water, and the number of individuals floating on the surface was counted.

Avoidance behaviour was expressed as Avoidance (A%):

(1) $$A\left(\%\right)=\frac{n_{c}-n_{t}}{n}\times 100$$

where n_c_ =  number of individuals located in the uncontaminated LUFA soil, n_t_ =  number of individuals located in the contaminated LUFA soils, and n = the total number of live individuals at the end of the experiment. Positive values indicate avoidance behaviour, and treatments with A ≥ 80% were considered as having “limited habitat functions” [46]. The avoidance test was considered validated if dual controls showed mortality of 10% and a stochastic distribution of the arthropods between the two soil sides.

The disaggregation effects were measured by the disaggregation index (DI), reflecting the extent of disaggregation, and the disaggregation-in-groups index (DG), reflecting its granularity:

(2) $$DI=\frac{s+2d}{n};\;DG=\frac{g}{n}$$

were s  =  number of groups with only 1 individual, d  =  number of groups with only 2 individuals, g = number of total groups formed at the end of the experiments, and n  =  the number of alive individuals at the end of the experiment. The index varies between 0/0.1 and 1, which represent the maximum degrees of aggregation and disaggregation, respectively, while 0.5 was fixed as the threshold level above which disaggregation affects 50% of the population. Further details regarding the index and validity criteria can be found in [47].

### 2.5. Statistical Analysis

Statistical analyses were conducted in R (v4.5.2) [48] using the packages dplyr, ggplot2, WRS2, boot, tidyr, gridExtra, and vegan. Data were tested for normality using the Shapiro-Wilk test and for homogeneity of variances using Levene’s test.

For avoidance data, the proportion of organisms on the uncontaminated soil at the end of the experiment (pc) was evaluated against a fixed reference value of 0.5 for each concentration using a robust one-sample test based on an M-estimator of central tendency [49]. In addition, 95% confidence intervals for pc at each concentration level were estimated using a non-parametric 10,000-bootstrap replicate to account for distributional uncertainty and potential deviations from normality. Compared to avoidance responses, which identify limited habitat conditions of treatment soils, the pc score is a more descriptive measure of the population’s distribution, providing additional information regarding the proportion of animals found on the contaminated and uncontaminated soil at the end of the bioassay.

Differences in response variable distributions, avoidance, and disaggregation indices among treatments were assessed using Kruskal–Wallis rank sum tests, as the data did not consistently meet the assumptions of parametric analyses. Dunn’s post hoc test, with Holm correction for multiple comparisons, was applied to identify which treatments differed significantly from the controls. To assess the degree of aggregation of the woodlice population, one-sample *t*-tests were performed, comparing the observed values of DI and DG with a fixed disaggregation threshold of 0.5, corresponding to a value at which 50% of the individuals are fragmented. This threshold was selected as a biologically meaningful reference value to distinguish between aggregated and dispersed population distributions within the experimental arenas.

Data were considered statistically significant for values of *p* < 0.05.

## 3. Results

Control bioassays conducted with only LUFA 2.2 soil confirmed the validity of the experimental setup for both populations, as organisms exhibited a stochastic spatial distribution between compartments, aggregation behaviour of at least 80% [47], and mortality below 10%, thus meeting standard acceptability criteria for avoidance tests [46].

The distinct physicochemical structures of LDPE and PP led to different product release profiles. The detailed list of the compounds detected in LDPE and PP (aldehydes, ketones, dicarboxylic compounds, alcohols, polyols, carboxylic acids, polyoxygenated compounds, aliphatic hydrocarbons) is reported in the Supplementary Materials (Table S1).

### 3.1. Behavioural Responses of Soil Arthropods Exposed to Leachates of Pristine and Aged LDPE

**Figure 2:**
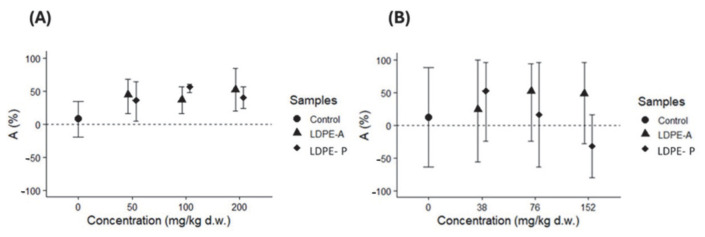
Avoidance response (%) of *Folsomia candida* (**A**) and *Porcellionides pruinosus* (**B**) induced by leachates of pristine (P) and aged (A) LDPE polymers at the selected concentrations (mg/kg d.w.). Data are reported as mean ± standard deviation (SD), based on 10 individuals per treatment and 5 replicates per treatment (*n* = 50; r = 5).

Regarding the behavioural responses of *F. candida*, overall results of the proportion of individuals (pc) against a fixed reference value of 0.5 significantly deviated among treatments (χ^2^ = 13.083, df = 6, *p* = 0.0417), indicating a treatment-dependent redistribution of organisms (Table 1). However, the avoidance percentage did not differ significantly across treatments (Kruskal–Wallis χ^2^ = 7.068, df = 6, *p* = 0.3146), indicating that, despite a measurable redistribution of individuals between compartments, exposure to LDPE leachates did not result in an alteration in soil habitat functionality (Figure 2A).

For the *P. pruinosus* population, the overall results of the proportion of individuals (pc) showed a highly significant treatment effect (χ^2^ = 29.338, df = 6, *p* = 5.25 × 10^−5^), indicating strong behavioural modulation in response to LDPE leachates (Table 1). Conversely, avoidance percentages (A%) were not significantly different among treatments (Kruskal–Wallis χ^2^ = 3.912, df = 6, *p* = 0.6885), suggesting a trend similar to that observed for *F. candida* (Figure 2B).

Inspection of treatment-specific proportions in springtail bioassays revealed that the highest deviation was observed for LDPE-P at 100 mg/kg d.w. (pc = 0.78), followed by LDPE-A at 200 mg/kg d.w. (pc = 0.76), indicating a marked redistribution of organisms (Table S2). Furthermore, avoidance responses, with estimated 95% CI based on 10,000 bootstrap replicates, exhibited a dose-dependent increase for both polymer types, increasing from 44% (50 mg/kg d.w.) to 52% (200 mg/kg d.w.) in LDPE-A, whereas LDPE-P elicited a peak response at 100 mg/kg d.w. (56%; 95% CI: 48–60) (Table S3).

Regarding the woodlice bioassay, treatment-specific proportions (Table S2) revealed a monotonic increase in pc for LDPE-A (from 0.62 to 0.78 at 76 mg/kg d.w., with a slight decrease to 0.738 at 152 mg/kg d.w.), whereas LDPE-P showed a non-linear response, with a marked decrease at the highest concentration (pc = 0.34 at 152 mg/kg d.w.), suggesting a different behavioural response to aged versus pristine leachates. Avoidance percentages supported the pattern of the proportional test but were characterised by wide confidence intervals (Table S3), likely attributable to the gregarious behaviour of *P. pruinosus* [47], which inflates variance and reduces the precision of bootstrap estimates. For LDPE-P at 152 mg/kg d.w., a negative mean avoidance (−32%; 95% CI: −76 to 16) was observed, while LDPE-A treatments generally yielded positive mean avoidance values, ranging from 24% (95% CI: −56 to 100) to 52% (95% CI: −24 to 96).

**Figure 3:**
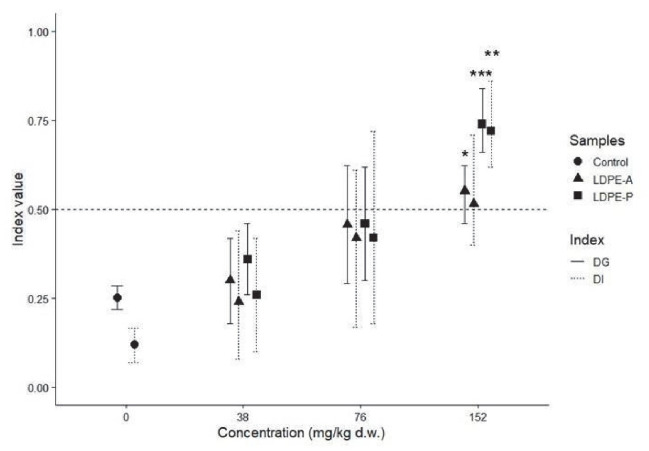
Disaggregation index (DI) and disaggregation in groups index (DG) of *P. pruinosus* population induced by leachates of pristine (P) and aged (A) LDPE polymers at the selected concentrations (mg/kg d.w.). Data are reported as mean ± standard deviation (SD), based on 10 individuals per treatment and 5 replicates per treatment (*n* = 50; r = 5). Significance codes of *p* value: 0 ‘***’ 0.001 ‘**’ 0.01 ‘*’ 0.05.

In contrast to the avoidance responses, the disaggregation index (DI) and the disaggregation in group index (DG) were significantly affected by experimental factors (Figure 3), including group identity (control vs. LDPE), dose, and their interaction (Table 2). At the group level, both DI (χ^2^ = 10.16, *p* = 0.0062) and DG (χ^2^ = 10.21, *p* = 0.0061) showed significant differences only at the highest concentrations, indicating influences of different ageing conditions of leachates. Similarly, concentration had a strong effect on both indices, with DI (χ^2^ = 18.41, *p* = 0.00036) and DG (χ^2^ = 20.18, *p* = 0.00016) exhibiting even higher levels of statistical significance, suggesting a dose-response sensitivity of woodlice. Importantly, the interaction between aging and concentration was statistically significant (χ^2^ = 19.55, *p* = 0.0033).

Pairwise comparisons based on Dunn’s test revealed significant differences between the highest concentration and the control condition. Specifically, DI values were significantly higher at 152 mg/kg d.w. in LDPE-P (Z = −3.928, *p* = 0.00180) compared to the control. Similarly, DG values were significantly increased at the same concentration in both LDPE-P (Z = −4.069, *p* = 0.00099) and LDPE-A (Z = −2.805, *p* = 0.0352) relative to the control.

One-sample *t*-tests against the disaggregation threshold of 0.5 (i.e., where 50% of the woodlice population is fragmented) revealed a clear aggregation pattern in control conditions, with both indices significantly lower than the threshold (DG: t_4_ = −13.80, *p* < 0.001; DI: t_4_ = −14.70, *p* < 0.001). Confidence intervals for both indices did not overlap 0.5, confirming strong aggregation.

In LDPE-P treatments, only at 152 mg/kg d.w. were both indices significantly higher than the threshold level (DG: t_4_ = 4.71, *p* = 0.009; DI: t_4_ = 2.99, *p* = 0.040), with confidence intervals entirely above 0.5, indicating a significant shift toward disaggregation at the highest concentration. For LDPE-A, a significant aggregation was detected only for DG at 38 mg/kg d.w. (t_4_ = −2.83, *p* = 0.047), while for the other concentrations neither index differed significantly from the threshold (*p* > 0.05), suggesting a reduced impact of LDPE on the aggregation behaviour of woodlice after aging (Table S4).

### 3.2. Behavioural Responses of Soil Arthropods Exposed to Leachates of New and Aged PP

**Figure 4:**
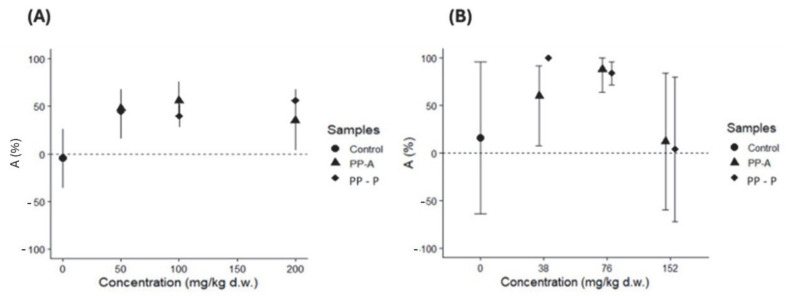
Avoidance response (%) of *F. candida* (**A**) and *P. pruinosus* (**B**) induced by leachates of pristine (P) and aged (A) PP polymers at the selected concentrations (mg/kg d.w.). Data are reported as mean ± standard deviation (SD), based on 10 individuals per treatment and 5 replicates per treatment (*n* = 50; r = 5).

Overall results of the proportion test (pc) of individuals of *F. candida* significantly deviated among treatments (χ^2^ = 23.614, df = 6, *p* = 0.000614), indicating redistribution of organisms among treatments (Table 3). However, the avoidance percentage (A%) of collembolans did not differ significantly across treatments (Kruskal–Wallis χ^2^ = 10.086, df = 6, *p* = 0.1211) (Figure 4A). Similarly, for the *P. pruinosus* population (Table 3), an overall strong significant distribution of individuals among treatments was observed by the proportion test in response to PP leachates (χ^2^ = 94.83, df = 6, *p* = 2.2 × 10^−16^), but such redistribution was not related to a significant reduction in soil habitat functions (Figure 4B) (Kruskal–Wallis χ^2^ = 11.101, df = 6, *p* = 0.0853).

Treatment-specific proportions of springtails indicated that the highest deviation was observed at 100 mg/kg d.w. for PP-A (pc = 0.78) and at 200 mg/kg d.w. for PP-P (pc = 0.78), while intermediate values were recorded at lower concentrations (Table S5). In terms of avoidance responses, mean values rose from 44% at 50 mg/kg d.w. (95% CI: 16–68) to 52% at 200 mg/kg d.w. (95% CI: 20–84) in PP-A, with an intermediate reduction at 100 mg/kg d.w. (36%; 95% CI: 16–56) (Table S6). A similar non-linear trend was observed for PP-P, with avoidance peaking at 100 mg/kg d.w. (56%; 95% CI: 48–60), followed by a decrease at the highest concentration (40%; 95% CI: 24–56).

The population of woodlice displayed a more pronounced and treatment-dependent response. Under PP-A exposure, proportions increased with concentration up to 76 mg/kg d.w. (pc = 0.94), followed by a decline at the highest dose (pc = 0.56 at 152 mg/kg), while PP-P treatments elicited a strongly non-monotonic pattern, with net response at lower concentrations (pc = 1.00 at 38 mg/kg d.w.; 0.92 at 76 mg/kg d.w.) and a reduction in avoidance at the highest concentration (pc = 0.32 at 152 mg/kg d.w.) (Table S5). Similarly, avoidance responses increased sharply up to 76 mg/kg (88%; 95% CI: 64–100) in PP-A treatments, followed by a pronounced decline at 152 mg/kg d.w. (12%; 95% CI: −60 to 84), while non-monotonic avoidance responses emerged for PP-P, ranging from 100% at 38 mg/kg d.w. to 4% (95% CI: −72 to 80) at the highest concentration (Table S6).

**Figure 5:**
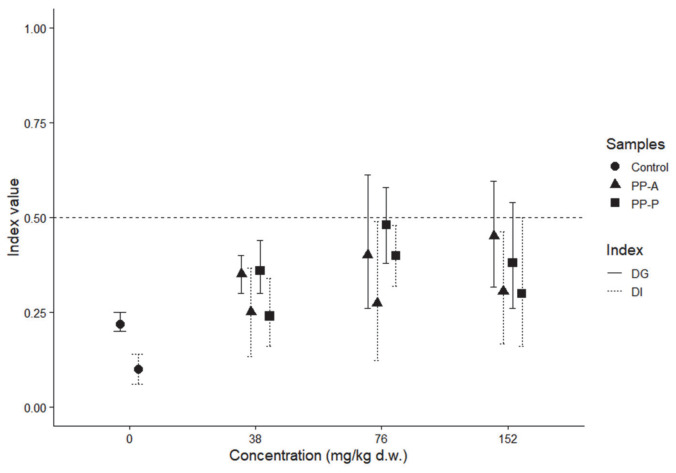
Disaggregation index (DI) and disaggregation in groups index (DG) of *P. pruinosus* population induced by leachates of pristine (P) and aged (A) PP polymers at the selected concentrations (mg/kg d.w.). Data are reported as mean ± standard deviation (SD), based on 10 individuals per treatment and 5 replicates per treatment (*n* = 50; r = 5).

The disaggregation index (DI) and the disaggregation in group index (DG) were significantly affected by experimental factors (Figure 5), including group identity (control vs. PP), dose, and their interaction (Table 4).

Pairwise comparisons based on Dunn’s test revealed significant differences between treatments and the controls. For DI, a significant increase was observed only in PP-P at 76 mg/kg d.w. (Z = 3.76, *p* = 0.00351) compared to the control. In contrast, DG showed a broader response, with significant increases detected in PP-P at 76 mg/kg d.w. (Z = 3.58, *p* = 0.00708) and in PP-A at 152 mg/kg d.w. (Z = 3.31, *p* = 0.0198) relative to the control.

One-sample *t*-tests against the disaggregation threshold of 0.5 revealed a consistent aggregation pattern in control conditions, with both indices showing values significantly lower than expected under the disaggregation threshold (DG: t_4_ = −21.00, *p* < 0.001; DI: t_4_ = −19.00, *p* < 0.001). Mean values (DI = 0.100; DG = 0.220) and their confidence intervals did not overlap the threshold, confirming strong aggregation in unexposed woodlice populations.

In PP-P treatments, significant deviations from the threshold were observed only at the lowest concentration (38 mg/kg d.w.) for both indices (DG: t_4_ = −3.50, *p* = 0.025; DI: t_4_ = −5.10, *p* = 0.007), indicating enhanced aggregation relative to the disaggregation threshold.

A similar but weaker pattern at 38 mg/kg d.w. was observed in PP-A treatments, where both indices were significantly lower than the threshold (DG: t_4_ = −5.20, *p* = 0.014; DI: t_4_ = −3.87, *p* = 0.031). At higher concentrations, neither DI nor DG differed significantly from 0.5 (*p* > 0.05) in both pristine and aged PP, suggesting that the disaggregation indices, although differing from the control condition, did not significantly exceed the 0.5 threshold value (Table S7).

## 4. Discussion

This study investigated the effects of raw micronized granules leachates comparable to additive-free pre-production products to obtain useful insights to discern the role of polymer leachate itself in MP toxicity. The outcomes highlight the role of polymer types in the onset of plastic toxicity, suggesting that weathering may modulate behavioural responses depending on polymer type and endpoint.

In light of these findings, noteworthy topics are discussed below in three different paragraphs:

1. sensitivity of the behavioural bioassays;
2. role of polymer type;
3. plastic aging status.

### 4.1. Sensitivity of the Behavioural Bioassays

This study evaluated the sensitivity of behavioural alterations induced by MP leachates in two different edaphic species. To the best of our knowledge, there are few studies that cope with the effects of polymer leachates on soil organisms compared to the aquatic systems, making a comprehensive comparison difficult. The only other study that has assessed the toxicity of aged and pristine plastics on terrestrial arthropods showed no significant avoidance response to PP and LDPE [50]. However, the authors did not take into consideration the effect of plastic leachates.

In the present study, neither *F. candida* nor *P. pruinosus* exhibited avoidance responses, suggesting a similar impact between particles [50] and leachates. In terms of responsiveness, both springtails and woodlice showed a complementary pattern of avoidance responses, in line with [51,52]. In terms of variability, avoidance responses in woodlice were characterised by extremely wide confidence intervals compared to springtails (Table S3). This high variability is likely attributable to the discrete and clustered spatial distribution of individuals, driven by the intrinsic gregarious behaviour of *P. pruinosus* [47]. Consequently, clustered responses may reduce the predictability of avoidance responses, particularly where social behaviour influences movement and habitat selection of soil organisms [53]. In contrast, the evaluation of disaggregation behaviour of woodlice allowed to obtain useful data on the potentially higher hazard of LDPE than PP. The results herein suggest that bioassays involving terrestrial isopods may be more informative and sensitive due to the gregarious nature of woodlice, which amplifies responses that are not captured by the conventional avoidance test [47]. Further studies are needed to confirm the observed behavioural alterations, involving more replicates to enhance the reliability and statistical significance of the results. In terms of ecological consequences, the loss of aggregation behaviour leads to a functional impairment at the population level, exposing individuals to dehydration and reducing fitness [54]. Therefore, the use of this novel endpoint as a potential indicator of functional impairment in woodlice populations may be proposed to complement standard ecotoxicological bioassays.

### 4.2. The Role of Polymer Type

The polymer type plays an important role in the onset of plastic leachate toxicity [55,56,57]. However, most studies evaluated the effects of commercial plastic products that contained complex mixtures of toxic chemicals and additives. The results of this study suggest that even the free-additive polymer may influence the toxicity of leachates, with LDPE proving stronger behavioural alterations than PP at the highest tested concentration under the tested conditions. To the best of our knowledge, this is the first evidence of the hazard of leachates derived from additive-free MPs on terrestrial organisms.

Similar evidence from tests on cell-based bioassays and aquatic organisms has been reported in the literature. Leachates from raw pre-production polymers (PE, PP, PS) induced oxidative stress responses in cell-based bioassays [58]. In a subsequent study, the same authors reported that the leachates from additive-free MPs of PE reduced algae biomass, cell growth, and photosynthetic activity, while leachates from other polymers (PET, PP, PS) did not show detectable ecotoxicological effects on the same endpoints [25]. A study conducted on the marine copepod *Nitocra spinipes* (Crustacea) showed that new pristine PE and PP pellets which contained low levels of additives produced leachates with toxicity as high as or even higher than commercial materials (with additives) of the same polymer type [59]. The main outcome of these studies [25,58,59] was that the toxicity of additive-free pre-production plastic leachates was caused by polymer degradation products from artificial weathering.

In the present study, it is suggested that the intrinsic chemical reactivity of LDPE and PP may have contributed to the observed differences in behavioural endpoints, in light of the release in leachates of different compounds (aldehydes, ketones, dicarboxylic compounds, alcohols, polyols, carboxylic acids, polyoxygenated compounds, aliphatic hydrocarbons) (Table S1). Further studies are required to better investigate the role of these compounds in the onset of leachate toxicity. In this perspective, one possible approach could be to test the effects of the compound alone and in mixtures and to better clarify their interaction with the tested organisms.

### 4.3. Plastic Aging Status

Weathering processes can enhance the leaching of additives from parent plastic materials, potentially influencing MPs toxicity [60]. At the same time, the discussion of the impact of weathering on MP hazard is still ongoing due to the presence of contrasting outcomes.

In an exploratory screening study with *N. spinipes*, the leachate hazard varied under simulated weathering, with toxicity of aged leachate that can be equal, less, or more than the pristine one [61]. Contrastingly, different evidence suggested that the aging regime does not affect MP toxicity [18,25,50], while others showed that aged plastic leachate induces lower effects than pristine ones [62,63]. In this context, the results of this study suggest that leachate from pristine LDPE can lead to a greater disaggregation than that from aged LDPE in woodlice, while neither the pristine nor the aged PP plastics appear to cause behavioural alterations. It is therefore suggested that artificial weathering may modulate behavioural responses depending on polymer type and endpoint. The distinct photo-oxidative degradation pathways of LDPE and PP may potentially have played a role in the outcomes observed in this study. In particular, it has been demonstrated that PP is subject to faster radical initiation and oxidative chain scission due to the presence of tertiary carbon atoms in the polymer backbone [33,34]. In contrast, LDPE typically exhibits a more protracted and incremental oxidation process, characterised by the gradual accumulation of oxygenated functionalities [37,38]. It is hypothesised that these differences may have contributed to the observed outcomes. In particular, PP produced larger amounts of low-molecular-weight oxygenated compounds (such as aldehydes, ketones, organic acids, and oxidised hydrocarbons), while LDPE showed higher relative amounts of long-chain alkanes (Table S1). Terrestrial isopods are known to possess highly specialised chemosensory systems associated with antennal sensilla and cephalic sensory structures [54]. Comparative neuroanatomical studies in Oniscidea demonstrated substantial adaptations of the olfactory system during terrestrial evolution, including restructuring of deutocerebral and tritocerebral neuropils [64]. In addition, electrophysiological evidence obtained in *Armadillidium vulgare* showed that terrestrial isopods can detect chemical extracts through porous sensilla and apical gustatory organs [65]. Behavioural avoidance approaches for *P. pruinosus* have already been successfully applied to soil contaminants and traffic-derived particles, including benzothiazole-containing tire particles, highlighting the sensitivity of this species to chemically complex contaminant mixtures [47,66]. Similarly, *F. candida* possesses a well-developed chemosensory apparatus with porous chemoreceptive sensilla on antennal segments and a postantennal organ as a specialised sensory structure connected to the protocerebrum [67,68]. Behavioural studies further demonstrated that *F. candida* is capable of detecting and responding to volatile organic compounds produced by fungi and other environmental sources [69], and recent evidence suggests that sesquiterpenes may act as repellents for this species [70].

Taken together, these findings suggest the hypothesis that the different responses observed in the present study may be partially driven by the difference in the volatile and semi-volatile degradation products released from aged plastics, particularly among a more apolar profile compared to a profile represented by a large prevalence of oxygenated compounds. The proposed mechanistic explanations remain hypothetical because no direct link between specific compounds and behavioural responses has been investigated. In this context, given the complexity of the leachate mixtures and the absence of compound-specific exposure data for most detected chemicals, the present interpretation should be considered preliminary and hypothesis-generating. In this context, further studies are essential to better understand the principal factors that have caused the more pronounced disaggregation behaviour observed for pristine LDPE treatment compared to aged LDPE.

## 5. Conclusions

In terrestrial ecosystems, plastic leachates are the result of a complex mixture of polymers, varying in size, shape, surface functionalities, additive composition, and degree of weathering. The specific hazards inherent in each polymer need to be identified in order to facilitate the identification of the most hazardous one for the purpose of risk management. In this perspective, this study investigated the role of additive-free leachates derived from two common plastic polymers in agricultural soils, LDPE and PP, in eliciting behavioural alterations in *Folsomia candida* and *Porcellionides pruinosus*. Overall, neither polymer leachate induced significant avoidance responses, suggesting a limited contribution of leachate-mediated toxicity to potential depopulation effects. In contrast, disaggregation assays in woodlice revealed a potential influence of plastic-derived leachates on social behaviour, with LDPE eliciting a significant effect. Photo-oxidative weathering emerged as an important modulating factor, highlighting the role of environmental ageing processes in shaping behavioural responses to plastic-derived chemicals. Notably, alterations in gregariousness were observed exclusively at the highest tested concentration (152 mg/kg d.w.), representative of heavily contaminated soil scenarios. In line with these outcomes, it is recommended to conduct further studies to investigate the effects of leachates on terrestrial organisms due to the potential for such alterations to impact the functional impairment of the woodlice population. Further research is therefore warranted to elucidate the long-term effects of plastic leachates on terrestrial invertebrates. Future studies should investigate lower and environmentally realistic concentrations through chronic exposure assays, while also incorporating additional ageing scenarios, including variations in UV spectra, duration of atmospheric exposure, and temperature regimes. Comparative assessments between additive and additive-free plastics, as well as interactions with co-occurring stressors (e.g., pesticides and fertilisers), will require further study for a more ecologically realistic evaluation of plastic-associated risks in terrestrial ecosystems.

## Figures and Tables

**Table 1:** Overall statistical results referred to the proportion (pc) and avoidance responses (A%) of individuals of *Folsomia candida* and *Porcellionides pruinosus* exposed to leachates of LDPE. The table reports the statistical χ^2^ and *p* value. Significance codes of *p* value: 0 ‘***’ 0.001 ‘*’ 0.05.

Model Organisms	Response Variable	Test	χ^2^	df	*p* Value
*Folsomia candida*	Proportion (pc)	χ^2^ test for equality of proportions	13.083	6	0.0417 *
	Avoidance (A%)	Kruskal–Wallis test	7.068	6	0.3146
*Porcellionides pruinosus*	Proportion (pc)	χ^2^ test for equality of proportions	29.338	6	5.25 × 10^−5^ ***
	Avoidance (A%)	Kruskal–Wallis test	3.912	6	0.6885

**Table 2:** Overall statistical results referred to the disaggregation index (DI) and disaggregation in groups index (DG) of individuals of *Porcellionides pruinosus* exposed to leachates of LDPE polymers. The table reports the χ^2^ and *p* value. Significance codes of *p* value: 0 ‘***’ 0.001 ‘**’ 0.01.

Disaggregation Indices	Factors	χ^2^	*p*-Value
DI	Interaction	19.55	0.0033 **
	Groups	10.16	0.0062 **
	Dose	18.41	0.00036 ***
DG	Interaction	21.62	0.0014 **
	Groups	10.21	0.0061 **
	Dose	20.18	0.00016 ***

**Table 3:** Overall statistical results referred to the proportion (pc) and avoidance responses (A%) of individuals of *F. candida* and *P. pruinosus* exposed to leachates of PP. The table reports the statistical χ^2^ and *p* value. Significance codes of *p* value: 0 ‘***’ 0.001.

Model Organisms	Response Variable	Test	χ^2^	df	*p* Value
*Folsomia candida*	Proportion (pc)	χ^2^ test for equality of proportions	23.614	6	0.0006148 ***
	Avoidance (A%)	Kruskal–Wallis test	10.086	6	0.1211
*Porcellionides pruinosus*	Proportion (pc)	χ^2^ test for equality of proportions	94.83	6	2.2 × 10^−16^ ***
	Avoidance (A%)	Kruskal–Wallis test	11.101	6	0.0853

**Table 4:** Overall statistical results referred to the disaggregation index (DI) and disaggregation in groups index (DG) of individuals of *P. pruinosus* exposed to leachates of PP polymers. The table reports the χ^2^ and *p* value. Significance codes of *p* value: 0 ‘***’ 0.001 ‘**’ 0.01.

Disaggregation Indices	Factors	χ^2^	*p*-Value
DI	Interaction	17.04	0.0091 **
	Groups	13.82	0.0010 **
	Dose	14.33	0.0025 **
DG	Interaction	18.84	0.0044 **
	Groups	16.09	0.00032 ***
	Dose	16.54	0.00088 ***

## Data Availability

Data is contained within the article or Supplementary Materials.

## Supplementary Materials

The following supporting information can be downloaded at: https://www.mdpi.com/article/10.3390/toxics14060502/s1. Section S1: “Leachate characterization procedure” [19,71,72]; Table S1: list of the compounds detected in LDPE (pristine and aged) and PP (pristine and aged) leachates; Table S2: values of the proportion test (pc) for each treatment and concentration of the avoidance test in individuals of *Folsomia candida* and *Porcellionides pruinosus* exposed to leachates of pristine (P) and aged (A) LDPE polymers; Table S3: avoidance (%) responses of individuals of *Folsomia candida* and *Porcellionides pruinosus* exposed to leachates of new (N) and aged (A) LDPE polymers. Values are reported as mean and 95% confidence intervals (CI) estimated using a non-parametric 10,000 bootstrap replicates; Table S4: statistical *t*-test results against a threshold value of 0.5 referred to the disaggregation index (DI) and disaggregation in groups index (DG) of individuals of *Porcellionides pruinosus* exposed to leachates of pristine (P) and aged (A) LDPE polymers. The table reports the t statistic and *p* value; Table S5: values of proportion test (pc) for each treatment and concentration of the avoidance test in individuals of *Folsomia candida* and *Porcellionides pruinosus* exposed to leachates of pristine (P) and aged (A) PP polymers; Table S6: Avoidance (%) responses of individuals of *Folsomia candida* and *Porcellionides pruinosus* exposed to leachates of pristine (P) and aged (A) PP polymers. Values are reported as mean and 95% confidence intervals (CI) estimated using a non-parametric 10,000 bootstrap replicates; Table S7: Statistical *t*-test results against a threshold value of 0.5 referred to the disaggregation index (DI) and disaggregation in groups index (DG) of individuals of *Porcellionides pruinosus* exposed to leachates of pristine (P) and aged (A) LDPE polymers. The table reports the t statistic and *p* value.

## Abbreviations

The following abbreviations are used in this manuscript:

MP	Microplastic
LDPE	Low-density polyethylene
PP	Polypropylene
LDPE-P	Pristine LDPE
LDPE-A	Aged LDPE
PP-P	Pristine PP
PP-A	Aged PP
DOC	Dissolved organic carbon
HS-SPME	Solid-phase microextraction
WHC	Water holding capacity
GC-MS	Gas chromatography–mass spectrometry
pc	Proportion
A	Avoidance
DI	Disaggregation index
DG	Disaggregation in group index
