# Supplementary material for: Effects of Pristine and Aged LDPE and PP Microplastic Leachates on Behavioural Responses of the Soil Arthropods Folsomia candida and Porcellionides pruinosus
Source: Toxics. 2026 Jun 9;14(6):502. doi: 10.3390/toxics14060502 (PMC13307738; doi:10.3390/toxics14060502)
Supplement: Supplementary file 1 [file toxics-14-00502-s001.zip › toxics-4321750-supplementary.pdf]

toxics

Supplementary Materials of the Article entitled

Effects of pristine and aged LDPE and PP microplastic leachates on behavioural responses of the soil arthropods *Folsomia candida* and *Porcellionides pruinosus*

Andrea Masseroni[1], Lorenzo Federico[1], Alessandro Becchi[1], Maurizio Quinto[2], Francesco Saliu[1] and Sara Villa[1*]

[1] DISAT, Department of Earth and Environmental Sciences, University of Milano-Bicocca, Piazza della Scienza 1, Milan 20126, Italy
[2] DAFNE, Department of Agriculture, Food, Natural Resources and Engineering, University of Foggia, Via Napoli 25, Foggia 71122, Italy
* Correspondence: sara.villa@unimib.it
A.M. and L.F. contributed equally.

Section S1. Leachate characterization procedure

Analytical verification was performed on the compounds detected in the leachates. Leachates were divided into 3 aliquots and submitted to dissolved organic carbon (DOC), extractable and Semi-volatile and Volatile Organic Compounds (SVOC/VOCs) determination. A semi-quantitative approach was adopted. One representative model compound was selected for each chemical class, and calibration curves were generated for these reference compounds. It was then assumed that compounds belonging to the same chemical class exhibited comparable instrumental response factors. Therefore, the analytical quantification of the leachates should be interpreted as semi-quantitative rather than fully quantitative.

A1. DOC analysis.

In order to quantify the total amount of the organic species released and dissolved into the aqueous phase, 10 mL aliquots of the leachate solutions were filtered and transferred into 20 mL glass vials by using 10 mL polypropylene Luer syringes (VWR, USA) attached to a filter (25 mm, 0.22 µm) and analysed by employing a total organic carbon analyser, TOC- VCSH (Shimadzu, Japan), configured in liquid mode. The data collected from the leachates and the calibration curves were then used to extrapolate DOC release rates per cumulative UV dose (mg MJ^{-1}) by adapting an approach published elsewhere [1]. Before analysis, all samples, blanks, and calibrants were acidified with hydrochloric acid and purged with ultrapure air to ensure the elimination of residual contamination of inorganic carbon. System calibration was obtained by processing seven calibration points from 0.003 to 0.300 mg/L, prepared by diluting a potassium hydrogen phthalate carbon standard in acidified Milli-Q water. Calibration curves showed a linear response in the tested range, with $r^2 = 0.991$. The resulting limit of quantification was 0.014 mg/L.

A2. GC-MS analysis of extractable

5 mL aliquots of the filtered leachates were transferred into a 10 mL glass test tube, added with an internal standard (naphthalene deuterated from Sigma-Aldrich at 50 ng/mL), vigorously stirred for 10 min, and then extracted 3 times with Dichloromethane (2 mL, fraction I) and ethyl acetate (2 mL, fraction II). These organic extracts were collected separately in vials and concentrated under a nitrogen stream down to 0.2 mL, then re-diluted to 1 mL for the final injection into the GC–MS system. Before analysis, the ethyl acetate extracts were subjected to trimethylsilylation with 100 μL of *N*, *O*-bis(trimethylsilyl)trifluoroacetamide with 1 % trymethylchlorosilane (BSTFA +1% TMCS, ≥ 99 %, Sigma-Aldrich) for 2 h at 60. Milli-Q water control samples and procedural blanks (run with distilled water) were analysed using the same procedure in order to ensure quality control. GC–MS analyses were carried out by employing an Agilent 8860 instrument equipped with a 5977B mass-selective detector. The injector was operated in split mode at 280 °C and with a 20:1 ratio. Analytes were separated with a DB5 -5MS UI capillary column (30 m × 0.250 mm, film thickness 0.25 μm, Agilent Technologies, USA) using the following temperature program: 5 min at 50 °C, 10 °C/min to 320 °C for 5 min. Gas carrier (He) at 1.2 mL/min and purge flow 3.0 mL/min. Scan Speed 0.1 amu/s. Scan rate 40–500 amu. Deconvolution and feature alignment were obtained by using the deconvolution algorithm tools available on MzMine 3 open software [2] for GC-EI, applying hierarchical clustering, maximum retention time tolerance set up at 0.5 min, and the minimum number of signals in a pseudo spectrum set up at 6. The tentative identification of the chemical species was based on a comparison with mass spectra and retention times of standard compounds previously analysed, applying the same chromatographic conditions and mass-selective detector parameters using the cosine algorithm. For the identification of the unknown compounds displaying no match with our in-house reference data set, we referred to the NIST Mass Spectral Library (v.14) by using the related spectral research algorithm. A positive match was assumed when the match factor was >80 and the calculated Kovats index resulted within 15% of the values reported in the literature.

A3. HS-GC/MS of SVOC/VOC fraction

Volatiles and semi-volatiles released from both pristine and photo-aged granules were profiled by employing headspace solid phase microextraction (HS-SPME) followed by GC–MS. In this case, 5 mL aliquot of the leachate was placed in a 10 mL headspace glass vial and capped. The headspace microextraction was then carried out using an 85 μm carboxen/polydimethylsiloxane (DVB/CAR/PDMS) fibre mounted on a solid phase micro-extraction (SPME) device (Supelco, JVA Analytical Ltd., Ireland). Fibres were thermally conditioned following the manufacturer's recommendations before their first use. The samples were held in a thermal equilibration phase at 40 °C for 10. Fibres were then directly inserted into the sample headspace and exposed for 30 min at 40 °C and finally desorbed into the GC injection port. Analysis was carried out by using an Agilent 7890 A GC–MS coupled with an Agilent 5975C mass-selective detector (Little Falls, DE, USA). Injections were made in splitless mode using an SPME injection sleeve (0.75 mm I·D) at 250 °C for 350 s. Chromatographic separations were obtained by using an Agilent DB-Wax column (60 m × 0.25 mm I.D., 0.25 μm film thickness, *J*&W Scientific, Folsom, CA, USA). The carrier gas flow rate (Helium, 99.999 %) was 1.0 mL/min. The oven temperature was initially set at 45 °C for 6 min, then increased to 240 °C at a rate of 4 °C/min, and kept at 240 °C for 5 min, before returning to the initial temperature. The MS detector was operated in scan mode (mass range 25–350 amu). Acquisition and data processing were performed by MSD Agilent ChemStation (Little Falls, DE, USA).

A4. Compound identification

Compound identifications in both the dissolved and VOC/SVOC fractions were performed by comparing the collected mass spectra to those of the National Institute of Standards and Technology database (NIST/EPA/NIH Mass Spectral Library with Search Program, data version NIST 05, software version 2.0d). Identity confirmation occurred only if the minimum requirement of a match score higher than 80 was met. Moreover, to obtain unambiguous identifications, for each identified compound from database searching, the Kovats retention index was calculated. Then the molecular assignments were accepted when the difference between the experimental linear retention index (LRI), based on a homologous series of n-alkanes, and the reference value was lower than 10%. The concentration of each identified chemical species was estimated semi-qualitatively by using the calibration curve of the standard reference compound of the same chemical class. Data were then presented by considering total counts for chemical classes (compounds aggregated based on the occurrence of a specific functional group). More details of the procedure are available in [3].

Table S1. List of the compounds detected in LDPE (pristine and aged) and PP (pristine and aged) leachates.

Aldehydes				
Compound	LDPE pristine	LDPE aged	PP pristine	PP aged
Formaldehyde		X		X
Acetaldehyde		X		X
Propanal		X		X
Butanal		X	X	X
Hexanal		X	X	X
Heptanal		X		X
Octanal		X	X	X
Nonanal		X	X	X
Decanal				X
Tetradecanal				X
acrolein				X
Benzaldehyde				X
**Ketones and dicarboxylic compounds**				
**Compound**	**LDPE pristine**	**LDPE aged**	**PP pristine**	**PP aged**
2,4-DIMETHYLCYCLOPENTANONE			X	X
2,4-Pentanedione				
2,5-Hexanedione		X		X
2,5-Octanedione				X
2-Butanone		X		X
2-Decanone				X
2-Dodecanone		X		X
2-Heptanone		X		X
2-Hexanone				X

2-Nonanone			X	X
2-Octanone		X	X	X
2-Pentanone				X
2-Pentanone, 4-hydroxy-4-methyl-			X	
2-Undecanone		X		
3,6-Heptanedione			X	X
3-Heptanone			X	X
3-Hexanone				X
3-Hexanone, 5-methyl-				X
3-HYDROXY-3-METHYL-2-PENTANONE		X		X
3-Nonanone				X
3-Octanone				
3-Penten-2-one, 4-methyl-				X
4-Octanone				X
5-Decanone				X
5-METHYL-2-HEXANONE				X
5-Nonanone				X
Acetone		X		X
CYCLOPROPYL METHYL KETONE				X
2-METHYL-2-PENTEN-4-ONE			X	X

Alcohols and polyols				
**Compound**	**LDPE pristine**	**LDPE aged**	**PP pristine**	**PP aged**
(S)-(+)-5-Methyl-1-heptanol				X
1,2-Ethanediol, diacetate			X	X
1-Butanol		X	X	X
1-Heptanol, 2,4-diethyl-			X	X

1-Hexadecanol			X	X
1-Octanol, 3,7-dimethyl-			X	X
1-Propanol			X	X
10-METHYL-1-DODECANOL				X
2,5-DIMETHYL-2,5-HEXANEDIOL				X
2,5-Hexanediol, 2,5-dimethyl-				X
2-Isopropyl-5-methyl-1-heptanol				X
3-PENTANOL			X	X
DIETHYLENE GLYCOL			X	X
Ethyl alcohol		X	X	X
Isopropyl alcohol				X
1-Pentanol		X		X
1-Hexanol		X		X
4-Penten-2-ol			X	X
3,6-NONADIENOL		X		X

**Carboxylic acid**				
**Compound**	**LDPE pristine**	**LDPE aged**	**PP pristine**	**PP aged**
Acetic acid		X	X	X
Formic acid		X		X
Isobutyric acid			X	
Propanoic acid		X		X
Propanoic acid, 2-methyl-				X
Butanoic acid		X	X	X
Butanoic acid, 2-methyl-				X
Pentanoic acid				X
Pentanoic acid, 4-oxo-				X
Hexanoic acid			X	X
Hexanoic acid, 2-ethyl-				X

Heptanoic acid				X
Octanoic acid		X		X
Nonanoic acid				X
3-Pentenoic acid, 4-methyl-				X
2-Propenoic acid, 2-methyl-				X

Polyoxigenated compounds				
Compound	LDPE pristine	LDPE aged	PP pristine	PP aged
3,4-DIHYDRO-2H-PYRAN				X
3-Acetyl-2,5-dimethyl furan			X	X
Gluconolactone				X
2-Propanone, 1-hydroxy-				X
1-Hydroxy-2-butanone		X		X
2-Pentanone, 4-hydroxy-4-methyl-		X		X
2-Butanone, 4-hydroxy-		X		X
Ethanone, 1-cyclopropyl-				X
Oxirane, heptadecyl-				X
1-HYDROXY-2-PROPANONE			X	X
5-Methylfurfuryl acetate			X	X
propryl acetate				X

Aliphatic hydrocarbons				
Compound	LDPE pristine	LDPE aged	PP pristine	PP aged
1-Dodecene	X		X	
1-HEPTADECENE			X	
1-TETRADECENE			X	
1-Tridecene	X		X	
pentadecene	X		X	
1-Undecene			X	

2,2,4,6,6-PENTAMETHYLHEPTANE		X	
2,2,4-TRIMETHYLPENTANE		X	
2,4-Dimethyl-1-heptene		X	
2-Nonene, (E)-	X		
2-Pentene, (Z)-	X		
3-Ethyl-3-methylheptane		X	
4-METHYLHEPTANE		X	
CIS-2-OCTENE		X	
Cyclodecane	X		
Cyclododecane	X		
Decane, 3,7-dimethyl-	X		
Docosane	X	X	
Dodecane	X	X	
Dodecane, 2-methyl-	X		
Dodecane, 4-methyl-	X	X	
Eicosane	X		
Heneicosane	X	X	
Heptadecane		X	X
Heptadecane, 2-methyl-		X	
HEPTANE	X		
Heptane, 2,2,4,6,6-pentamethyl-		X	
Heptane, 2,4-dimethyl-		X	
Heptane, 2,4-dimethyl-		X	
2,4-Dimethyl-1-heptene		X	
Hexacosane	X		
Hexadecane	X	X	
Hexadecane, 7,9-dimethyl-		X	
Hexane	X		

Hexane, 2,2-dimethyl-			X	
Hexane, 3,3-dimethyl-			X	
Hexane, 3-ethyl-			X	
Nonane, 2,6-dimethyl-			X	
NORBORNANE	X			
Octadecane	X		X	
OCTANE	X		X	
Octane, 5-ethyl-2-methyl-			X	
PENTACOSANE	X		X	
Pentadecane	X		X	X
Pentadecane, 2,6,10,14-tetramethyl-	X		X	
Pentadecane, 2-methyl-			X	
Tetracosane	X			
Tetradecane	X			
Tetradecane, 2-methyl-			X	
Tridecane	X		X	
UNDECANE	X		X	
Undecane, 2-methyl-			X	
Undecane, 5,7-dimethyl-		X	X	

Table S2. Values of the proportion test (pc) for each treatment and concentration of the avoidance test in individuals of *Folsomia candida* and *Porcellionides pruinosus* exposed to leachates of pristine (P) and aged (A) LDPE polymers.

Model organisms	Treatment	Concentration (mg/kg d.w.)	Proportion (pc)
Folsomia candida	Control	0	0.54
	LDPE-P	50	0.68
		100	0.78
		200	0.70
	LDPE-A	50	0.72
		100	0.68
		200	0.76
Porcellionides pruinosus	Control	0	0.56
	LDPE-P	38	0.76
		76	0.58
		152	0.34
	LDPE-A	38	0.62
		76	0.78
		152	0.74

Table S3. Avoidance (%) responses of individuals of *Folsomia candida* and *Porcellionides pruinosus* exposed to leachates of new (N) and aged (A) LDPE polymers. Values are reported as mean, and 95% confidence intervals (CI) estimated using a non-parametric 10,000 bootstrap replicates.

Model organisms	Treatment	Concentration (mg/kg d.w.)	Mean A (%)	95% CI lower	95% CI upper
Folsomia candida	Control	0	8	-20	34
	LDPE-P	50	36	4	64
		100	56	48	60
		200	40	24	56
	LDPE-A	50	44	16	68
		100	36	16	56
		200	52	20	84
Porcellionides pruinosus	Control	0	12	-64	88
	LDPE-P	38	52	-24	96
		76	16	-64	96
		152	-32	-76	16
	LDPE-A	38	24	-56	100
		76	52	-24	94
		152	47	-28	95.6

Table S4. Statistical t-test results against a threshold value of 0.5 referred to the disaggregation index (DI) and disaggregation in groups index (DG) of individuals of *Porcellionides pruinosus* exposed to leachates of pristine (P) and aged (A) LDPE polymers. The table reports the t statistic and p value.

**Sample**	**Concentration (mg/kg)**	**Index**	**Mean ± CI (95%)**	**df**	**t**	**p-value**
Control	0	DG	0.253 (0.213–0.293)	4	-13.80	<0.001***
		DI	0.122 (0.064–0.180)	4	-14.70	<0.001***
LDPE-P	38	DG	0.360 (0.193–0.527)	4	-2.33	0.080
		DI	0.260 (0.003–0.517)	4	-2.59	0.061
	76	DG	0.460 (0.203–0.717)	4	-0.43	0.688
		DI	0.420 (-0.023–0.863)	4	-0.50	0.642
	152	DG	0.740 (0.598–0.882)	4	4.71	0.009**
		DI	0.720 (0.516–0.924)	4	2.99	0.040*
LDPE-A	38	DG	0.300 (0.104–0.496)	4	-2.83	0.047*
		DI	0.240 (-0.046–0.526)	4	-2.53	0.065
	76	DG	0.458 (0.190–0.726)	4	-0.44	0.685
		DI	0.420 (0.078–0.762)	4	-0.65	0.551
	152	DG	0.552 (0.425–0.679)	4	1.13	0.320
		DI	0.516 (0.258–0.774)	4	0.17	0.872

Table S5. Values of proportion test (pc) for each treatment and concentration of the avoidance test in individuals of *Folsomia candida* and *Porcellionides pruinosus* exposed to leachates of pristine (P) and aged (A) PP polymers.

Model organisms	Treatment	Concentration (mg/kg d.w.)	Proportion (pc)
Folsomia candida	Control	0	0.48
	PP-P	50	0.72
		100	0.70
		200	0.78
	PP-A	50	0.74
		100	0.78
		200	0.67
Porcellionides pruinosus	Control	0	0.58
	PP-P	38	1.00
		76	0.92
		152	0.32
	PP-A	38	0.80
		76	0.94
		152	0.56

Table S6. Avoidance (%) responses of individuals of *Folsomia candida* and *Porcellionides pruinosus* exposed to leachates of pristine (P) and aged (A) PP polymers. Values are reported as mean, and 95% confidence intervals (CI) estimated using a non-parametric 10,000 bootstrap replicates.

Model organisms	Treatment	Concentration (mg/kg d.w.)	Mean A (%)	95% CI lower	95% CI upper
Folsomia candida	Control	0	8	-20	34
	PP-P	50	36	4	64
		100	56	48	60
		200	40	24	56
	PP-A	50	44	16	68
		100	36	16	56
		200	52	20	84
Porcellionides pruinosus	Control	0	16.0	-64	96
	PP-P	38	100.0	100	100
		76	84.0	72	96
		152	4.0	-72	80
	PP-A	38	60.0	8	92
		76	88.0	64	100
		152	12.0	-60	84

Table S7. Statistical t-test results against a threshold value of 0.5 referred to the disaggregation index (DI) and disaggregation in groups index (DG) of individuals of *Porcellionides pruinosus* exposed to leachates of pristine (P) and aged (A) LDPE polymers. The table reports the t statistic and p value.

Sample	Concentration (mg/kg)	Index	Mean ± CI (95%)	df	t	p-value
Control	0	DG	0.220 (0.200–0.240)	4	-21.00	<0.001 ***
		DI	0.100 (0.080–0.120)	4	-19.00	<0.001 ***
PP-P	38	DG	0.360 (0.250–0.470)	4	-3.50	0.025 *
		DI	0.240 (0.150–0.330)	4	-5.10	0.007 **
	76	DG	0.480 (0.200–0.760)	4	-0.34	0.749
		DI	0.400 (0.100–0.700)	4	-2.24	0.089
	152	DG	0.380 (0.180–0.580)	4	-1.39	0.235
		DI	0.300 (0.080–0.520)	4	-1.91	0.129
PP-A	38	DG	0.300 (0.180–0.420)	4	-5.20	0.014 *
		DI	0.200 (0.080–0.320)	4	-3.87	0.031 *
	76	DG	0.401 (0.150–0.652)	4	-0.95	0.394
		DI	0.274 (0.050–0.498)	4	-2.04	0.111
	152	DG	0.452 (0.250–0.654)	4	-0.62	0.570
		DI	0.306 (0.090–0.522)	4	-2.22	0.090

References

1. Delre, A.; Goudriaan, M.; Morales, V.H.; Vaksmaa, A.; Ndhlovu, R.T.; Baas, M.; Keijzer, E.; De Groot, T.; Zeghal, E.; Egger, M.; Rockmann, T.; Niemann, H.. Plastic photodegradation under simulated marine conditions. Mar. Pollut. Bull., **2023**, *187*, 114544.
2. Schmid, R.; Heuckeroth, S.; Korf, A. Integrative analysis of multimodal mass spectrometry data in MZmine 3. *Nat. Biotechnol.*, **2023**, *41*, 447–449.
3. Isa, V.; Saliu, F.; Quinto, M.; Becchi, A.; Veronelli, M.; Spadaccino, G.; Lasagni, M.; Galli, P.; Lavorano, S. Impacts of micro-plastics on reef-building corals: Disentangling the contribution of the chain scission products released by weathering. *Sci. Total Environ.*, **2025**, *975*, 179239.

Disclaimer/Publisher's Note: The statements, opinions and data contained in all publications are solely those of the individual author(s) and contributor(s) and not of MDPI and/or the editor(s). MDPI and/or the editor(s) disclaim responsibility for any injury to people or property resulting from any ideas, methods, instructions or products referred to in the content.
